# *FANCD1/BRCA2* Plays Predominant Role in the Repair of DNA Damage Induced by ACNU or TMZ

**DOI:** 10.1371/journal.pone.0019659

**Published:** 2011-05-09

**Authors:** Natsuko Kondo, Akihisa Takahashi, Eiichiro Mori, Taichi Noda, Małgorzata Z. Zdzienicka, Larry H. Thompson, Thomas Helleday, Minoru Suzuki, Yuko Kinashi, Shinichiro Masunaga, Koji Ono, Masatoshi Hasegawa, Takeo Ohnishi

**Affiliations:** 1 Particle Radiation Oncology Research Center, Research Reactor Institute, Kyoto University, Osaka, Japan; 2 Advanced Scientific Research Leaders Development Unit, Gunma University, Gunma, Japan; 3 Department of Radiation Oncology, School of Medicine, Nara Medical University, Nara, Japan; 4 Department of Dermatology, School of Medicine, Nara Medical University, Nara, Japan; 5 Department of Molecular Cell Genetics, UMK Collegium Medicum, Bydgoszcz, Poland; 6 BBR Program, Lawrence Livermore National Laboratory, Livermore, California, United States of America; 7 Department of Genetics Microbiology and Toxicology, Stockholm University, Stockholm, Sweden; Genentech, United States of America

## Abstract

Nimustine (ACNU) and temozolomide (TMZ) are DNA alkylating agents which are commonly used in chemotherapy for glioblastomas. ACNU is a DNA cross-linking agent and TMZ is a methylating agent. The therapeutic efficacy of these agents is limited by the development of resistance. In this work, the role of the Fanconi anemia (FA) repair pathway for DNA damage induced by ACNU or TMZ was examined. Cultured mouse embryonic fibroblasts were used: *FANCA^−/−^*, *FANCC^−/−^*, *FANCA^−/−^C^−/−^*, *FANCD2^−/−^* cells and their parental cells, and Chinese hamster ovary and lung fibroblast cells were used: *FANCD1/BRCA2mt*, *FANCG^−/−^* and their parental cells. Cell survival was examined after a 3 h ACNU or TMZ treatment by using colony formation assays. All FA repair pathways were involved in ACNU-induced DNA damage. However, FANCG and FANCD1/BRCA2 played notably important roles in the repair of TMZ-induced DNA damage. The most effective molecular target correlating with cellular sensitivity to both ACNU and TMZ was *FANCD1/BRCA2*. In addition, it was found that *FANCD1/BRCA2* small interference RNA efficiently enhanced cellular sensitivity toward ACNU and TMZ in human glioblastoma A172 cells. These findings suggest that the down-regulation of *FANCD1/BRCA2* might be an effective strategy to increase cellular chemo-sensitization towards ACNU and TMZ.

## Introduction

High-grade gliomas pose a therapeutic challenge. Nimustine [1-(4-amino-2-methyl-5-pyrimidinyl) methyl-3-(2-chloroethyl)-3-nitrosourea; ACNU] and temozolo-mide (TMZ) are both DNA alkylating agents which are commonly used for chemo-therapy in the treatment of gliomas. In the past, nitrosourea drugs such as ACNU in Japan and central Europe, or carmustine [1,3-bis(2-chloroethyl)-1-nitrosourea; BCNU], in the United States were the standard of care in addition to radiation [1], [2]. This has changed since TMZ was shown to deliver benefits which were accompanied by lower levels of toxicity [3]. However, a recent meta-analysis also suggested the existence of a significant survival gain with the use of ACNU in newly diagnosed high-grade gliomas [4]. Even if these alkylating agents are used for the treatment for high-grade gliomas, the therapeutic efficacy of these agents is limited by the development of resistance to these agents, and the underlying mechanisms leading to the development of this resistance is still unknown.

ACNU and TMZ modify DNA at oxygen or nitrogen atoms in bases and oxygen atoms of phosphodiester bonds [5]. ACNU is a chloroethylating agent. The primary killing lesion appears to be the formation of *O^6^*-chloroethylguanine (G) [5]. This adduct is unstable, and undergoes intramolecular rearrangements leading to an intermediary *N^1^*-*O^6^*-ethenoG and, in a second step, to *N^1^*-G-*N^3^*-cytosine interstrand cross-links [5]. TMZ is classified as a monofunctional alkylating agent which modifies nucleotides through direct methylation, and the main killing lesion appears to be the formation of *O^6^*-methylG [5]. These two kinds of *O^6^*-alkylG adducts, *O^6^*-chloroethylG and *O^6^*-methylG, are repaired through the action of *O^6^*-methylG-DNA methyltransferase (MGMT) [5]. Other than MGMT, increasing evidence implicates the involvement of other kinds of repair pathways, for example, base excision repair (BER), Fanconi anemia (FA) repair, or double strand break (DSB) repair pathways which are involved in the repair of DNA damage induced by alkylating agents [6]–[11]. FA is characterized by developmental abnormalities, susceptibility to certain cancers, and sensitivity to DNA-DNA crosslinking agents [12]. Although the FA pathway was initially characterized in terms of the repair of DNA cross-linking agents [12], additional studies have disclosed an increasingly detailed involvement in general recombination repair and in the resolution of replication arrest [13]–[15]. In the regulation of the FA pathway, thirteen FA genes have been identified at present [12], but the exact mechanistic function of many of these FA proteins remains to be elucidated. FANCD1 has been identified as the breast cancer susceptibility protein BRCA2 [16] which regulates RAD51 in homologous recombination repair (HRR) [17]. The FA proteins (A, B, C, E, F, G, L, and M), together with the novel FA family members, FANCA associating Polypeptide (FAAP)24/100, are subunits of a nuclear core complex required for the mono-ubiquitylation of FANCD2 [18], [19]. It has been established that FANCD2 and other FA proteins, including FANCG, promote HRR [15], [20]–[22].

The work described here was designed to clarify which components in the FA repair pathways contribute significantly to ACNU and TMZ sensitivity. The activity of individual components of the FA repair pathway (FANCA, FANCC, FANCD1/BRCA2, FANCD2, and FANCG) leading to the repair of DNA damage induced by TMZ and ACNU was assessed using clonogenic survival assays. The cells used in this study formed a panel of mouse embryonic fibroblasts (MEF) and Chinese hamster ovary (CHO) and lung fibloblast cells defective in specific components in the FA repair pathways.

Next, to test whether the resulting observations were applicable to glioma cells, targeted repair pathways were down-regulated using small interference RNA (siRNA), and the sensitivity of human glioblastoma A172 cells to ACNU and TMZ was measured. DNA repair mechanisms which can be identified as contributing to ACNU and TMZ resistance should provide tools which could be applied to improve drug efficacy.

## Materials and Methods

### Cell lines

The fibroblasts from many kinds of knockout mouse of deficient in FANC genes (*FANCA^−/−^*, *FANCC^−/−^*, *FANCA^−/−^C^−/−^* and *FANCD2^−/−^* cells) and the wild-type mouse (*FANCwt*) [23] were obtained from the Fanconi Anemia Cell Repository, Oregon Health and Science Univ. (Portland, OR). The Chinese hamster lung fibroblast cell lines used in this study were: V79 (*FANCD1wt*), V-C8 (*FANCD1mutant* (*mt*)), V-C8+ *FANCD1* (*FANCD1revertant* (*rev*), V-C8 containing a BAC with the murine *FANCD1/BRCA2* gene) [24], [25]. The CHO cell lines used in this study were: AA8 (*FANCGwt*), KO40 (*FANCG^−/−^*), 40BP6 (*FANCGrev*, complemented with genomic CHO *FANCG*) [26], and SPD8 [27].

Human glioblastoma A172 cells were purchased from the American Type Culture Collection of Cell Cultures (Manassas, VA). All Cells were cultured in DMEM-10 [Dulbecco's modified Eagle's medium containing 10% (v/v) fetal bovine serum, 20 mmol/L 2-[4-(2-hydroxyethyl)-1-piperazinyl] ethanesulfonic acid, penicillin (50 units/mL), streptomycin (50 µg/mL), and kanamycin (50 µg/mL)]. The cells were cultured at 37°C in a conventional humidified CO_2_ incubator.

### Drug treatments

ACNU (Sigma Aldrich, Saint Louis, MO) was dissolved at a stock concentration of 10 mM in sterile H_2_O. ACNU stock solutions were stored at −20°C until used. TMZ (LKT Laboratories Inc. St. Paul, MN) was dissolved at a stock concentration of 100 mM in dimethylsulfoxide (DMSO). TMZ stock solutions were stored at −80°C until used. Cells were grown in medium containing ACNU or TMZ at various concentrations for 3 h, and then rinsed twice with PBS. At each different point, solution contains completely the same concentration of sterile H_2_O or DMSO except ACNU or TMZ.

### Cell survival

Cell survival was measured using a standard clonogenic survival assay as previously described [28]. The sensitivity of each cell line was assessed from its *D_50_* value, *i.e.* from the ACNU or TMZ dose which reduced cell survival to 50%. In order to accurately compare sensitivities to ACNU or TMZ in the repair defective cell lines, the relative *D_50_* values were normalized using the *D_50_* value of the parental cell lines.

### Recombination assays

SPD8 cells were grown in the presence of 5 µg/ml 6-thioguanine in order to reduce the frequency of spontaneous reversion prior to treatments. The protocol for the reversion assay [27] with SPD8 cells began with the inoculation of flasks (75 cm^2^) with 1.5×10^6^ cells in DMEM 4 h prior to a 24 h treatment with ACNU or TMZ in a 5% CO_2_ incubator. After treatment, the cells were rinsed three times with 10 ml of PBS, and 30 ml of DMEM was added to allow recovery for 48 h. The selection of revertants was performed by plating three dishes/group (3×10^5^cells/dish) in the presence of hypoxanthine-L-azaserine-thymidine (HAsT; 50 mM hypoxanthine, 10 mM L-azaserine, 5 mM thymidine). The cells were grown for 12 days before fixation with methanol, and then stained with a 2% Giemsa solution. For survival assay, about 500 cells per dish were plated on two dishes each and cultured 8 days. The cells were fixed and stained.

### RNA interference

The siRNA sequence used for human *FANCD1/BRCA2* was AAC AAC AAU UAC GAA CCA AAC UU [29]. The siRNA sequence of the non-specific negative control was the same as used previously [8]. The siRNA duplexes were synthesized and provided as a purified and annealed duplex by the Japan Bio Services Co., Ltd. (Saitama, Japan). Human *FANCD1/BRCA2* siRNA or a non-specific negative control siRNA was transfected into human glioblatoma A172 cells as previously described [8]. The siRNA sequences against the target *FANCD1/BRCA2* used here are the most popular in the previous reports. The cells were then trypsinized and plated for colony forming assays.

### Western Blotting

Total cellular protein amounts were quantified with a Bio-Rad protein assay kit (Bio-Rad Labs, Richmond, CA). Aliquots of proteins (20 µg) were subjected to Western blot analyses. Total cellular lysates were loaded onto 7% tris-glycine gels (Invitorogen), separated by electrophoresis at a constant voltage (125 V) and electro-transferred onto nitrocellulose membranes at 42 V. Membranes were blocked for 1 h at room temperature in blocking buffer (25 mM Tris pH 8.0, 125 mM NaCl, 1% Tween 20 [TBS-T buffer] containing 5% skim milk) and incubated with mouse monoclonal anti- FANCD1/BRCA2 antibody (Ab-4, Calbiochem and Oncogene, Calbiochem, Germany) or goat polyclonal anti-actin antibody (I-19; Santa Cruz Biotechnology, Santa Cruz, CA) for primary antibody for 2 h at room temperature. The membranes were washed with TBS-T buffer three times and incubated with a secondary antibody conjugated to horseradish peroxidase for 1 h. After washing three times, the blots were visualized by enhanced chemiluminescence method (GE Healthcare UK, Buckinghamshire, England) under the manufacturer's protocol. The amounts of the proteins in the samples were quantified by scanning profiles using the Scion imaging program (Scion, Frederick, MD). The relative ratio of the two bands to illustrate the intensity of FANCD1/BRCA2 protein expression is the average from triple experiments as measured by densitometry following β-actin normalization with and without *FANCD1/BRCA2* siRNA. The inserted photo is typical.

### MTS assay

A172 cells were plated into 96-well flat tissue-culture microplates (Falcon) at a density of 2×10^3^cells/50 µl/well and incubated at 37°C in 5% CO_2_ incubator. After a 6 h incubation, 50 µl of an ACNU or TMZ solution at a final concentrations of 50, 150, 300, or 500 µM, was added to individual wells, and the plates were incubated for an additional 3 days. On day 3, 20 µl of an MTS solution (CellTiter 96 Aqueous One Solution Cell Proliferation Assay; Promega Co., Madison, WI) was added to each well, and plates were incubated for a further 2 h following the manufacturer's protocol. The optical density at 492 nm was measured with a Microplate Reader (Multiskan FC; Thermo Fisher Scientific Inc., Waltham, MA). The results were shown as a percentage cell growth calculated as follows: % cell growth = (optical density of an ACNU-treated or TMZ-treated well−optimal density of cell-free control)/(optical density of cell-only control−optimal density of cell-free control)×100.

### Immunocytochemistry

Cells were grown on cover-glasses in 30 mm dish, fixed in 2% paraformaldehyde in PBS for 15 min at room temperature and washed in PBS. Then the cells were permeabilized for 5 min at 4°C in 0.2% Triton X-100, and blocked in PBS with 1% Bovine Serum Albumin (BSA) for 1 h at 37°C. Cells were then incubated with the rabbit polyclonal anti-Rad51 primary antibody (H-92, Santa Cruz) for 1 h at room temperature at 1∶300 dilutions in PBS containing 1% BSA, and washed three times in PBS containing 1% BSA for 10 min. The cells were incubated with Alexa Fluor 488-conjugated secondary antibody (Molecular Probes, Eugene, OR) for 1 h at room temperature at 1∶400 dilutions in PBS containing 1% BSA, and washed three times for 10 min in PBS. Cover-glasses were mounted at 1∶1000 dilutions of 4,6-diamidino-2-phenylindole. Fluorescence images were captured using a fluorescence microscope (Keyence, Tokyo, Japan) for analysis.

### Statistical analysis

Statistical analysis was performed using the Student's *t* test.

## Results

### Repair genes which respond to ACNU-induced DNA damage

**Figure 1:**
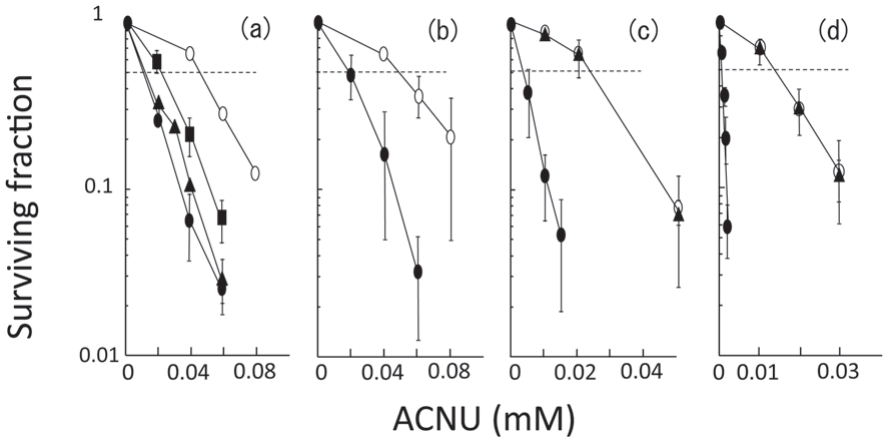
Sensitivity to ACNU. **a**, *FANCwt* cells (open circles), *FANCA^−/−^C^−/−^* cells (closed triangles), *FANCC^−/−^* cells (closed squares), and *FANCA^−/−^* cells (closed circles); **b**, *FANCwt* cells (open circles) and *FANCD2^−/−^* cells (closed circles); **c**, *FANCGwt* cells (open circles), *FANCGrev* cells (closed triangles), and *FANCG^−/−^* cells (closed circles); **d**, *FANCD1wt* cells (open circles), *FANCD1rev* cells (closed triangles), and *FANCD1mt* cells (closed circles). Each point represents the mean of three independent experiments; bars indicate the SD.

Cellular responses to ACNU were examined by using FA defective cells and their parental cells. As expected, all repair defective cells were more sensitive to ACNU (a cross-linking agent) than their parental cells (Fig. 1**a–d**). In particular, *FANCG^−/−^* and *FANCD1mt* cells exhibited a striking hypersensitivity to ACNU.

### Repair genes which respond to TMZ-induced DNA damage

**Figure 2 pone-0019659-g002:**
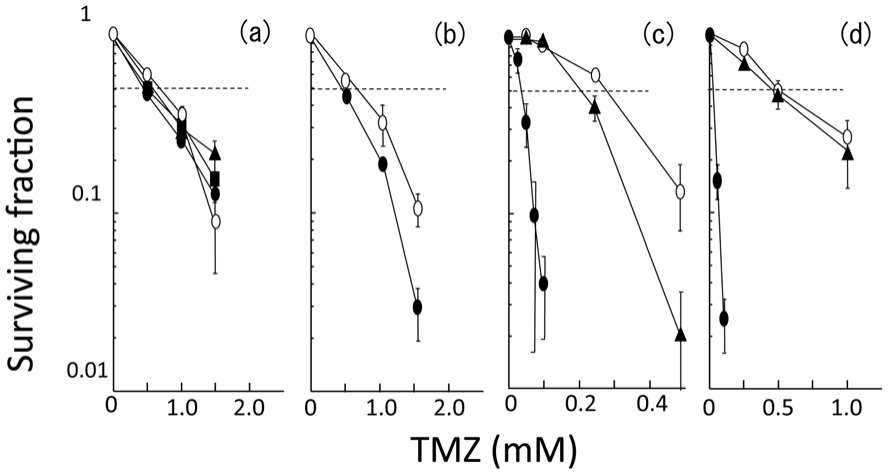
Sensitivity to TMZ. **a**, *FANCwt* cells (open circles), *FANCA^−/−^C^−/−^* cells (closed triangles), *FANCC^−/−^* cells (closed squares), and *FANCA^−/−^* cells (closed circles); **b**, *FANCwt* cells (open circles) and *FANCD2^−/−^* cells (closed circles); **c**, *FANCGwt* cells (open circles), *FANCGrev* cells (closed triangles), and *FANCG^−/−^* cells (closed circles); **d**, *FANCD1wt* cells (open circles), *FANCD1rev* cells (closed triangles), and *FANCD1mt* cells (closed circles). Each point represents the mean of three independent experiments; bars indicate the SD.

Cellular responses to TMZ were examined by using FA defective cells and their parental cells. The sensitivities to TMZ of *FANCA^−/−^*, *FANCC^−/−^*, and *FANCA^−/−^C^−/−^* cells were comparable to their parental cells (Fig. 2**a**). On the other hand, *FANCD2^−/−^*, *FANCD1mt*, and *FANCG^−/−^* cells were more sensitive to TMZ than their parental cells (Figs. 2**a–d**). As seen in the responses to ACNU, *FANCD1mt* and *FANCG^−/−^* cells exhibited a striking hypersensitivity to TMZ.

### Relative contributions of FA components to ACNU and TMZ sensitivity

**Figure 3:**
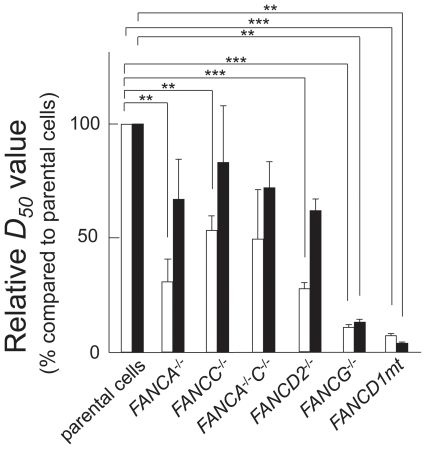
Relative *D_50_* values (% compared to proficient cells) for ACNU (open columns) and TMZ (closed columns). Two asterisks (**), *P*<0.01; three asterisks (***), *P*<0.001.

The relative *D_50_* values, listed sequentially in the order in which they increase (reflecting decreasing sensitivities to ACNU) are: *FANCD1mt* cells (0.06)<*FANCG^−/−^* cells (0.11)≪*FANCD2^−/−^* cells (0.27)≅*FANCA^−/−^* cells (0.31)<*FANCA^−/−^C^−/−^* cells (0.49)<*FANCC^−/−^* cells (0.53) (Fig. 3). The relative *D_50_* values listed in a similar manner for TMZ are: *FANCD1mt* cells (0.03)<*FANCG^−/−^* cells (0.12)≪*FANCD2^−/−^* cells (0.57)<*FANCC^−/−^* cells (0.67)≅*FANCA^−/−^C^−/−^* cells (0.71)<*FANCA^−/−^* cells (0.82) (Fig. 3).

From statistic analysis to ACNU, *P*<0.01 was between *FANCwt* and *FANCA^−/−^* or *FANCC^−/−^*; *P*<0.001 was between *FANCwt* and *FANCD2^−/−^*, *FANCwt* and *FANCG^−/−^*, *FANCwt* and *FANCD1mt*. To TMZ, *P*<0.01 was between *FANCwt* and *FANCG^−/−^*, *FANCwt* and *FANCD1mt*.

### Frequency of homologous recombination induced by ACNU or TMZ

**Figure 4:**
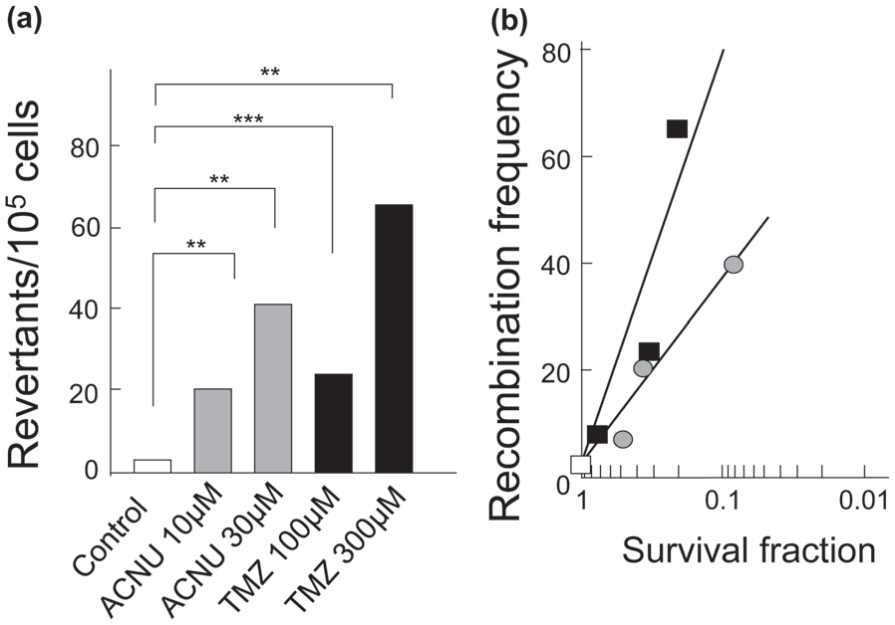
Homologous recombination in SPD8 cells. **a**, the reversion frequency from a non-functional to a functional *hprt* gene, giving resistance to HaST following a 24 h treatment with ACNU (10 µM and 30 µM, striped columns) or TMZ (100 µM and 300 µM, closed columns) is shown. Control, open column. Two asterisks (**), *P*<0.01; three asterisks (***), *P*<0.001. **b**, recombination frequency against the surviving fraction of cells following a 24 h treatment with ACNU (10 µM and 30 µM, striped circles) or TMZ (100 µM and 300 µM, closed squares). Control, open square.

Since HRR follows the FA repair pathway through the step involving FANCD1/BRCA2, it was of interest to know if HR in SPD8 cells was specifically induced by ACNU or TMZ. As a positive control, high HR frequencies were detected as 36.7 with Camptothecin (100 nM) with treatment for 24 h. In the case of ACNU and TMZ, HRR was obviously induced. After 10 µM and 30 µM ACNU treatments, the HR frequencies were 7.2-fold and 13.9-fold high, respectively, as compared with the control cells without ACNU treatment. After 100 µM and 300 µM TMZ treatments, on the other hand, the HR frequencies were 8.4-fold and 22.8- fold high, respectively (Fig. 4 **a**). We plotted the recombination frequency against the surviving fraction of SPD8 cells when exposed to varying concentrations of ACNU or TMZ. At 50% survival, HR frequencies with TMZ were about 2 times higher than those with ACNU (Fig. 4 **b**).

### Effect of *FANCD1/BRCA2* siRNA on ACNU or TMZ sensitivity in A172 glioblastoma cells

**Figure 5:**
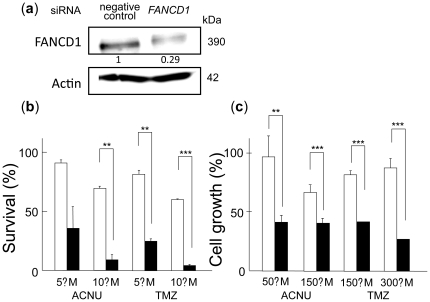
siRNA silencing of *FANCD1/BRCA2* in glioblastoma A172 cells after ACNU and TMZ treatments. **a**, expression analysis of FANCD1/BRCA2 in cells transfected with siRNA for human *FANCD1/BRCA2* or the negative control siRNA using Western blots. Actin was used as a loading control, and the relative ratios of protein were normalized using actin levels. **b**, effect of siRNA silencing of *FANCD1/BRCA2* on cellular sensitivity to ACNU and TMZ. **c**, effect of siRNA silencing of *FANCD1/BRCA2* on cell growth after ACNU or TMZ treatment. Open columns, negative control siRNA; closed columns, *FANCD1/BRCA2* siRNA. Columns show the mean of three independent experiments; the bars indicate the SD. An asterisk (*) indicates significant difference (*P*<0.05). Two asterisks (**), *P*<0.01; three asterisks (***), *P*<0.001.

To test whether this result was pertinent to chemotherapy agents used against glioblastomas, *FANCD1/BRCA2* expression was silenced using siRNA in A172 glioblastoma cells, and clonogenic survival assays and MTS assays were then performed with these cells. A172 cells were transfected with *FANCD1/BRCA2* siRNA for 48 h, and the expression of the protein was observed using Western blot analysis (Fig. 5**a**). The level of FANCD1/BRCA2 protein decreased to 29% when compared with the negative controls. After 5 µM and 10 µM ACNU treatments, *FANCD1/BRCA2* silencing caused a 64% and 90% reduction in colony formation when compared to cells transfected with the negative control siRNA (Fig. 5**b**). In addition, after a 5 µM or 10 µM TMZ treatment, *FANCD1/BRCA2* silencing caused 75% and 95% reductions in colony formation, respectively, when compared to cells transfected with the negative control siRNA (Fig. 5**b**). Similarly, after 50 µM and 150 µM ACNU treatments, *FANCD1/BRCA2* silencing caused a 57% and 38% reduction in cell growth when compared to cells transfected with the negative control siRNA (Fig. 5**c**). In addition, after 150 µM and 300 µM TMZ treatments, *FANCD1/BRCA2* silencing caused a 50% and 70% reduction, respectively in cell growth when compared to cells transfected with the negative control siRNA (Fig. 5**c**).

### Impaired Rad51 recombinase activity following *FANCD1/BRCA2* down-regulation

**Figure 6:**
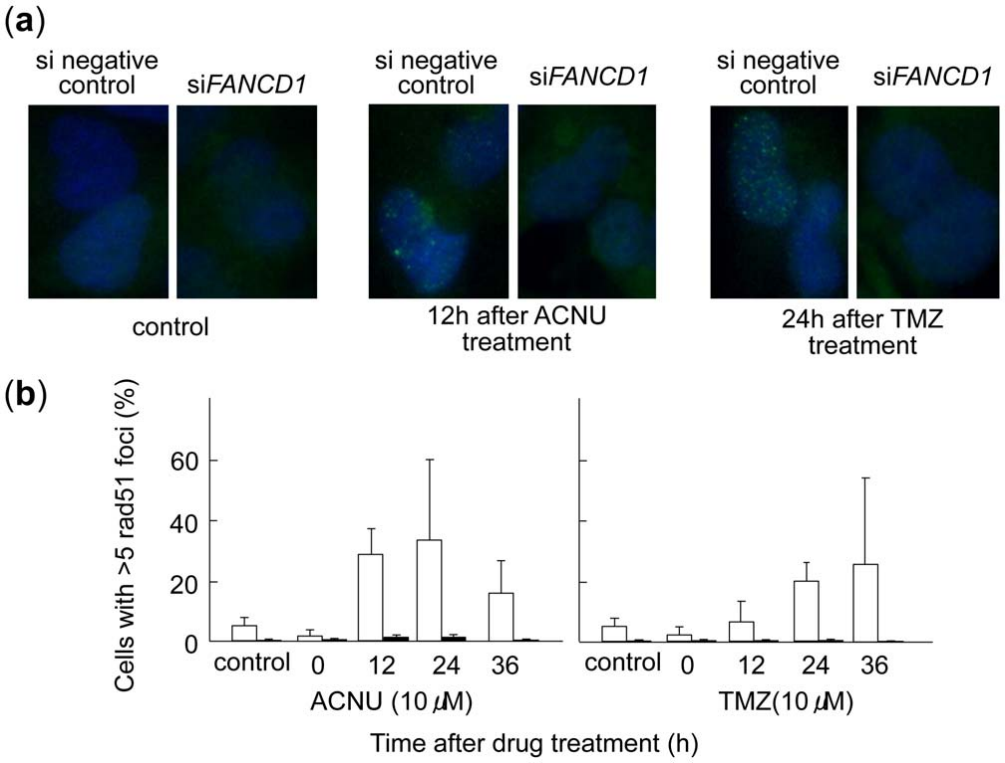
Rad51 foci formation in glioblastoma A172 cells after ACNU and TMZ treatments. **a**, typical photo. **b**, open columns, negative control siRNA; closed columns, *FANCD1/BRCA2* siRNA. Columns show the mean of two independent experiments; bars indicate the SD.

We detected apparent Rad51 foci in the nucleus of cells transfected with the negative control siRNA from 12 h to 36 h after ACNU or TMZ treatments. In contrast, we could not detect Rad51 foci in the cells transfected with *FANCD1/BRCA2* siRNA before and after ACNU or TMZ treatment. We, however, found Rad51 positive stain in cytoplasm alone (Fig. 6**a**). The number of cells with more than five Rad51 foci per nucleus was lower in the *FANCD1/BRCA2* siRNA transfected cells than the negative control siRNA transfected cells at 12 h, 24 h, and 36 h after ACNU or TMZ treatment (Fig. 6**b**).

## Discussion

**Figure 7:**
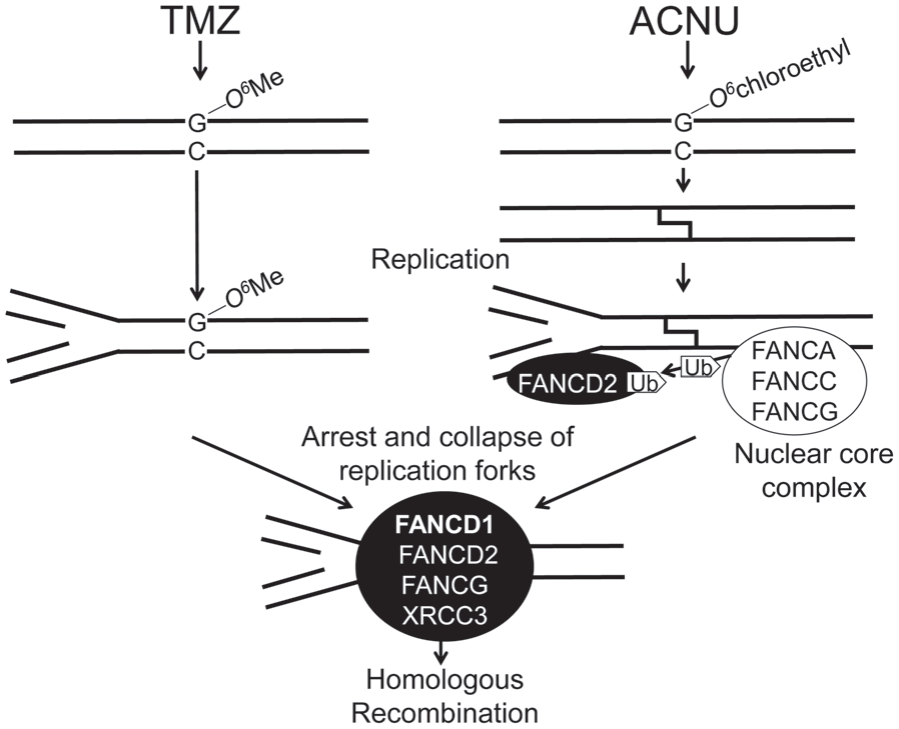
A schematic drawing showing a model of the repair for TMZ and ACNU induced DNA damage.

It has been previously shown that the FA pathway confers glioma resistance to DNA alkylating agents (TMZ and BCNU) and inhibition of FA pathway causes increased sensitivity to TMZ and BCNU [7]. Further, we examined in detail which components in the FA repair pathways contribute significantly to ACNU and TMZ sensitivity. Since the comparison of results between multiple cell lines of different species and different degrees of transformation may be worrisome part in the present manuscript, we compared each repair defective cell line at relative *D_50_* values (Fig. 3). At least, the genetic background of *FANCAwt*, *FANCCwt*, *FANCA/Cwt* and *FANCD2wt* were almost the same, therefore, it is definitely clear that ACNU-induced DNA-DNA cross-links might be repaired by FANCA, FANCC and FANCD2 which are components of FA nuclear core complex. This repair activity may function in similar manner as it does for the repair of damage caused by other cross-linking agents such as mitomycin C [12]. On the other hand, FANCA and FANCC might play a minor role in repair pathways for TMZ-induced DNA damage (Figs. 1–3), therefore this DNA damage is suggested to be repaired through nuclear core complex independent pathways. For both ACNU- and TMZ-induced DNA damage, FANCD2, and in particular, FANCG and FANCD1/BRCA2, play important roles (Figs. 1–3). These results are in agreement with previous studies which examined the sensitivity of these cells to other cross-linking agents (mitomycin C), methylating agents [methyl methanesulfonate (MMS)], or TMZ [11], [24], [26]. Recently, it was found that FANCD2 interacts with FANCD1/BRCA2 independently of the FA nuclear core complex [30]. In addition, FANCG has a role which is independent from its role in the FA nuclear core complex. Once FANCG is phosphorylated at Serine 7, it forms a complex comprising FANCD1/BRCA2-FANCD2-FANCG-XRCC3 (D1-D2-G-X3) which promotes HRR [30]. Other previous studies reported FA pathway promotes HRR [14], [15], [20]–[22]. In this study, following the notice that FANCD2, FANCG and FANCD1/BRCA2 might be involved in repair of both ACNU and TMZ (Figs. 1–3), it was also confirmed, using recombination assays, that the HRR pathway was active after ACNU and TMZ treatments (Fig. 4). If *O^6^*-methylG which is induced by TMZ and *O^6^*-chloroethylG which is induced by ACNU are not repaired by MGMT, they result in replication arrest and finally lead to DSBs. In case of TMZ, other DNA methylation, i. e. *N*-alkylation may also arrest replication forks as shown in MMS [31]. It is indicated that DSBs were formed in response to ACNU or TMZ by monitoring the expression of phosphorylated H2AX (γH2AX) [8]–[10]. Therefore it is assumed that the HRR activity was proposed as a repair mechanism for DSBs induced by ACNU or TMZ. It is suggested that FANCD1/BRCA2 for HRR contributes predominantly for repair of ACNU- and TMZ-induced DSBs (Fig. 3). From these results of this and other reports, a model for repair is suggested and a schematic drawing of this model is shown in Fig. 7.

It is possible that other FANC proteins which we didn't examine here play some roles in repair processes for these alkylating agents. For example, the *FANCF* deficient ovarian tumor cell line, 2008+vector cells exhibited an approximately a two- to three-fold decrease in survival as compared to the FA proficient 2008+*FANCF* cells to BCNU or TMZ [7]. FANCI is also monoubiquitinated based on nuclear core complex and interacts to form complex with FANCD2 [32]. The function of FANCI has not been well known yet, but it is supposed to play significant role in activation of FANCD2 and cooperate with FANCD2 in DNA repair. FANCJ which is identical with BRCA1-associating C-terminal helicase 1 (BACH1) or BRCA-interacting protein 1 (BRIP1) is supposed to associate with FA proteins or BRCA1 protein and function as a helicase for arrested replication fork independent of FA nuclear core complex [33]. Other FA nuclear complex proteins, for instance FANCL (an E3 ligase) which gives ubiquitination to FANCD2 [34], FANCM (a homologue of DNA helicase Hef in *E. coli*) [35] and, in addition, FAAP24 which interacts with FANCM [18] might affect repair for DNA damage induced by these alkylating agents. FANCN/partner of localizer of BRCA2 (PALB2) is expected to play as a critical role as FANCD1/BRAC2 in repair for DNA damage by ACNU or TMZ [36] independent of nuclear core complex.

It was shown that the siRNA for *FANCD1/BRCA2* gene down-regulated and increased the sensitivity of glioblastoma A172 cells to both ACNU and TMZ (Fig. 5**a, b**). *FANCD1/BRCA2* down-regulation impaired Rad51 foci formation in HRR (Fig. 6 **a**, **b**). Therefore, it is suggested that Rad51 function requires FANCD1/BRCA2 activity in the HRR for DNA damage induced with ACNU and TMZ. These results come to the conclusion that FANCD1/BRCA2 down-regulation could be a potentially useful strategy for enhancing the therapeutic effects of not only ACNU but also of TMZ, in treatments for glioma cells. Of course, when applied as a therapy, such chemo-sensitization must be delivered selectively to the tumor cells, since normal tissues could also depend on the same DNA repair pathway. Novel chemotherapeutic approaches utilizing loco-regional delivery have recently been shown to improve the survival of glioma patients [37]. Therefore, the combination of such a loco-regional delivery system and the simultaneous down-regulation of FANCD1/BRCA2 could be an effective tool to enhance chemotherapy results for glioma patients. The abnormalities of *FANCD1/BRCA2* gene have been reported in other cancer cells such as breast or ovarian cancer cells [12]. Therefore, ACNU and TMZ might be efficient for them. On the other hand, it is possible that nonhomologous end-joining repair might be predominant with error-prone under the depression of HRR after treatments with ACNU and TMZ in gliomas.
