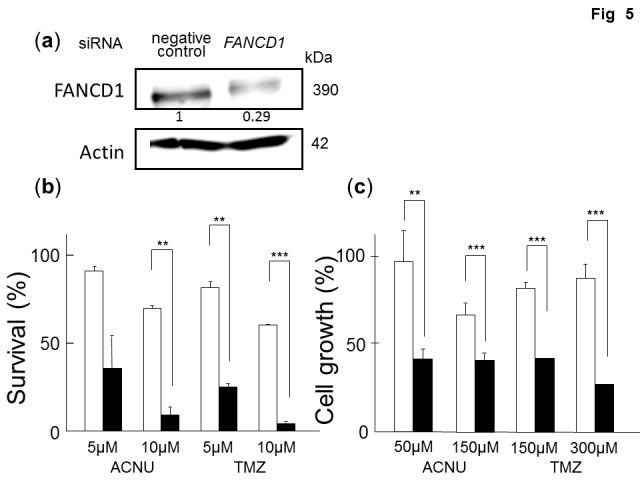# Correction: *FANCD1/BRCA2* Plays Predominant Role in the Repair of DNA Damage Induced by ACNU or TMZ

**DOI:** 10.1371/annotation/c6be24d1-bc23-43b4-ae01-b86dad174069

**Published:** 2011-06-10

**Authors:** Natsuko Kondo, Akihisa Takahashi, Eiichiro Mori, Taichi Noda, Małgorzata Z. Zdzienicka, Larry H. Thompson, Thomas Helleday, Minoru Suzuki, Yuko Kinashi, Shinichiro Masunaga, Koji Ono, Masatoshi Hasegawa, Takeo Ohnishi

In Fig. 5(b) and 5(c), the "question marks" under all columns should be changed to "micro." The correct Figure 5 can be viewed here: 

**Figure:**